# Differences in Autonomy and Health-Related Quality of Life between Resilient and Non-Resilient Individuals with Mild Cognitive Impairment

**DOI:** 10.3390/ijerph16132317

**Published:** 2019-06-30

**Authors:** Violeta Clement-Carbonell, Rosario Ferrer-Cascales, Nicolás Ruiz-Robledillo, María Rubio-Aparicio, Irene Portilla-Tamarit, María José Cabañero-Martínez

**Affiliations:** 1 Department of Health Psychology, Faculty of Health Science, University of Alicante, 03690 Alicante, Spain; 2 Department of Nursing, Faculty of Health Science, University of Alicante, 03690 Alicante, Spain

**Keywords:** mild cognitive impairment, resilience, coping, health-related quality of life, disability, dependence, autonomy, activities of daily living

## Abstract

The dramatic increase in the number of older people with Mild Cognitive Impairment (MCI) entails a serious public health problem. MCI involves different degrees of dependence that has been previously related to a decrease in Health-Related Quality of Life (HRQoL), due to impairment in the performance of activities of daily living. Resilient coping, as an adaptive coping style, could reduce the associated limitations derived by the characteristic deficits of MCI, and hence improve HRQoL. The principal objective of this work was to compare the level of autonomy (measured in terms of independence in the performance of basic (ADL) and instrumental (IADL) activities of daily living), and HRQoL between resilient and non-resilient individuals with MCI. The results showed a positive relationship between resilience, autonomy, and HRQoL. Hence, resilient participants exhibited higher independence in daily living activities and better HRQoL than non-resilient individuals. Mediation analyses confirmed an indirect influence of resilience on HRQoL through the mediation effect of better performance in IADLs. These findings underline the relevance of resilience as a coping style to compensate deficits in daily living in people with MCI. The inclusion of intervention programs, oriented to the promotion of resilience coping for older adults, might increase the autonomy levels in this population, improving their HRQoL.

## 1. Introduction

The dramatic increase in the number of older people with Mild Cognitive Impairment (MCI) entails a serious public health problem. MCI is an intermediate state of cognitive functioning between optimal cognitive status and clinical dementia, such as Alzheimer’s Disease (AD), showing a memory impairment beyond what is expected, taking into account the age and education of individuals [1,2]. The risk of progression to AD from MCI at an average annual rate is about 34.06% in clinical samples and 15.31% in community samples [3]. In this regard, Petersen et al. [4] found that 12% of persons with a diagnosis of MCI progress to AD over one year. Regarding the specific prevalence of MCI, Petersen and colleagues [5] suggested that around 16% of older people without dementia are affected by MCI. However, other studies estimated higher rates. For example, in a research carried out in the United States by Plassman et al. [6], it was estimated that 22% of subjects over 70 years suffer from MCI. Thus, it is seemed that cognitive impairment is a high-risk factor of increased disability and health deterioration among older adults [7]. 

Individuals with MCI suffer from some deficits that are considered determinants of their functional status, impairment, and dependence [8,9]. The MCI involves different degrees of dependence that has been previously related to a decrease in Health-Related Quality of Life (HRQoL), due to the impairment in the performance of activities of daily living [10,11]. Two types of everyday activities can be distinguished [12]. Basic activities of daily living (ADLs) such as bathing, eating, getting dressed, and mobility are often preserved in patients with MCI. However, instrumental activities of daily living (IADLs) are likely to deteriorate when cognitive abilities are impacted [13]. IADLs includes abilities related to the use of the telephone and transportation, shopping, cooking meals, handling money, taking care of medication, and other great number of everyday activities that require cognitive resources [14]. 

As has been indicated above, evidence from available research has demonstrated a significant reduction of autonomy in people with MCI, mainly due to impairment in the performance of activities of daily living. This loss of autonomy could be the main mechanism involved in the deterioration of HRQoL in people cognitively affected, as has been previously demonstrated [15]. A recent review which analysed the association between subjective cognitive impairment and HRQoL deterioration identified some specific difficulties that could contribute to these negative consequences for HRQoL, emphasizing the limitations in ADLs and IADLs as the main contributors to well-being and health impairment [16]. Hence, according to these results, in the study conducted by Woods et al. [17], cognitive impairment was only related to a decrease in quality of life when one or more ADLs were deteriorated. This fact points out the main role of loss of autonomy in people with MCI, a worsening of HRQoL. Individuals with MCI can experience frustration due to difficulties in completing these activities independently in their daily lives [18], suffering from high levels of anxiety and depression and dysfunctional social interactions [16], which could lead to the deterioration of HRQoL. This fact has been widely demonstrated, taking into account that various studies on HRQoL have found that older adults with MCI exhibit lower levels of well-being and higher levels of depression than their counterparts with normal aging [18,19]. In any case, the lack of autonomy in the performance of activities of daily living seems to be one of the most important mechanisms for the characteristic health impairment and quality of life reduction in people with MCI.

However, although the link between higher levels of dependence and HRQoL deterioration in people with MCI has been previously demonstrated, some individuals seems to cope effectively with the loss of autonomy derived from MCI, maintaining optimal levels of quality of life [15]. In this respect, the use of compensation strategies can help people with MCI improve their independence in everyday functioning. The results obtained by Tomaszewski et al. [20] in a recent study have shown that higher frequency of compensation strategies’ use was associated with higher levels of independence in daily life among older adults. Although compensation strategies could be an effective resource to face with specific deficits derived from MCI older adults, their implementation usually requires some creative thinking by the individual, which should find specific strategies oriented to the compensation of a certain deficit. Resilience, as a creative and positive coping style, could play a key role, having been traditionally associated with successful and healthy aging [21,22]. As proposed by reserve hypotheses [23], the link between resilience and healthy ageing is based on the compensation strategies that individuals implement to face with the characteristic physiological and cognitive deterioration during aging [24]. Taking into account that resilience can be considered as a coping style characterized by the use of compensative, creative and alternative strategies to face the deficits and adversities that people encounter in life, especially during aging, this fact acquires more relevance in older people with MCI. Consequently, resilient individuals could manage in a positive manner the negative consequences derived from MCI (e.g., memory loss, deficits in attention processing, etc.), through the development of compensation strategies [24], and hence, maintain adequate levels of autonomy and better HRQoL. However, to our knowledge, there is no prior research that has compared the levels of autonomy and HRQoL in resilient and non-resilient individuals with MCI. 

With all of this in mind, the main aim of this study was to identify differences in autonomy (measured in terms of independence in ADLs and IADLs) and HRQoL in resilient and non-resilient people with MCI. Moreover, the association between resilience, autonomy, and HRQoL, and the possible mediation effect of autonomy in the association between resilience and HRQoL were evaluated. Based on previous research, we hypothesized that resilience shows a positive association with higher levels of autonomy (both ADLs and IADLs) and better HRQoL [20]. Further, we expect to find significant differences in autonomy and HRQoL between resilient and non-resilient participants with MCI, as has been found in previous research [23]. Finally, although no previous studies have tested the mediation effect of autonomy in the relationship between resilience and HRQoL, we expect to find a significant mediation role of autonomy in this association. That is to say, an indirect effect of resilience on HRQoL through the mediation of autonomy (independence in ADLs and IADLs).

## 2. Materials and Methods 

### 2.1. Participants

The participants were selected by convenience sampling from community geriatric centers located in the city of Alicante (Spain). Inclusion criteria for the participants were: (1) being 50 years old or older, (2) being diagnosed with MCI, scoring in the range of 18 and 26 in the Spanish version of the Mini-Mental State Examination (MMSE) [25,26], and (3) being able to speak Spanish fluently. People with severe motor, sensory or psychiatric disorders were excluded from the study. The sample was composed by 62 individuals (66.1 % females; 33.9 % males) with ages from 60 to 97 years (mean age = 77.52 years; SD = 8.26), and with a mean MMSE of 22.91 (SD = 2.21). The sociodemographic characteristics of the whole sample and of each group separately, depending on resilience levels, are summarized in Table 1.

### 2.2. Instruments

#### 2.2.1. Brief Resilient Coping Scale (BRCS)

The Brief Resilient Coping Scale [27] was employed to asses resilience levels in the study sample. This questionnaire is configured by four items with a five-point Likert scale oriented towards the analysis of the ability of individuals to adaptively face stress. Based on the total score, individuals could be categorized as low-resilient (scored 13 or less), medium-resilient (scored 14 to 16) and high-resilient (scored 17 or more). The Spanish version has shown adequate validity and reliability scores, with Cronbach alpha of 0.86 [28]. In this study, the internal consistency of the BRCS was 0.60. 

#### 2.2.2. The Short Form-12 Health Survey (SF-12)

HRQoL was evaluated with the Spanish version of the Short Form Healthy Survey questionnaire (SF-12) [29]. This instrument, previously validated for the Spanish population, is composed of 12 items, which evaluate eight domains: general health, physical functioning, role physical, role emotional, body pain, social functioning, mental health and vitality. Based on these eight dimensions, two summary components were obtained: physical component summary (PCS) and mental component summary (MCS). The items are coded on a scale of 1 (‘worse health condition’) to 100 (‘best health condition’). Scores equal to or less than 30 are considered a risk. The SF-12 is a reliable and valid tool for the measurement of HRQoL, showing adequate validity and reliability scores [29]. In the current sample, the internal consistency of this instrument was 0.83.

#### 2.2.3. Barthel Index

To evaluate the level of participants’ dependence on ADLs, the Spanish version of the Barthel Index was employed [30]. This instrument analyzes the levels of individuals´ autonomy in the performance of 10 daily activities (personal toilet, bathing, feeding, getting on and off the toilet, ascending and descending stairs, dressing, controlling bowel and bladder). Higher scores on the Barthel Index indicate less dependency (0–20 totally dependent, 21–60 severely dependent, 61–90 moderately dependent, 91–99 slightly dependent or 100 independent). In the present study, the internal consistency of this instrument was 0.81.

#### 2.2.4. The Lawton Instrumental Activities of Daily Living

The Spanish version of The Lawton Instrumental Activities of Daily Living (IADL) Scale [31] was used to evaluate the functional deterioration of participants in instrumental activities of daily living. This instrument evaluates eight domains of IADLs: ability to use telephone, shopping, food preparation, housekeeping, laundry, mode of transportation, responsibility for own medications, and ability to handle finances. Persons are scored according to their highest level of functioning in each category. A summary score ranges from 0 (low function, dependent) to 8 (high function, independent). Higher scores are indicative of more independency. In the present study, the internal consistency was 0.86.

### 2.3. Procedure

Prior to conducting the study, the centers were contacted to inform them about the aim of the study and its procedure, inviting them to participate. The study was approved by the Vice Presidency and Ministry of Equality and Inclusive Policies of the Valencian Government (cod. 9631). The battery of questionnaires was administered in an interview format by an expert psychologist in the geriatric assessment field between May and October 2018. In these interviews, the researcher collected general information and administered a battery of self-reported questionnaires for evaluating resilience, HRQoL, and independence in ADLs and IADLs. Prior to the start of the study, a pilot study was carried out in a small sample with the aim of checking the understanding of the items, viability, and administration procedure. The present study was carried out according to the guidelines of the Declaration of Helsinki and the European Union Good Clinical Practice Standards. To protect confidentiality and anonymity of the data, codes were assigned to identify the participants. All participants read the information document with the objectives of the study and signed an informed consent to participate.

### 2.4. Data Analysis

Descriptive analyses of the sociodemographic characteristics and cognitive functioning were carried out. Participants were divided into two groups adapting the original cut-offs (low-resilient individuals scored 13 or less, medium-resilient 14 to 16 and high resilient individuals scored 17 or more) proposed by Sinclair and Wallston [27]. In the present study, the group of “non-resilient” individuals (*n* = 32) was composed of participants scoring 13 or less, the ‘low-resilient’ group in the original study. On the other hand, the group of “resilient” individuals (*n* = 30) was formed by individuals scoring 14 or more (the groups of medium and high resilience individuals in the original study). Pearson’s correlations were applied to test the relationships between resilience, ADLs, IADLs and HRQOL. The differences between resilient and non-resilient groups in each domain of ADLs, IADLs and HRQOL were analysed using the Student´s t test for independent samples. To test the mediation effect of levels of dependence, measured by ADLs and IADLs, on the relationship between resilience and HRQOL, multiple mediation analyses were conducted controlling for age, sex, marital status and cognitive functioning. Concretely, two models were tested for each summary component of the HRQOL (Physical Health Summary and Mental Health Summary). For mediation analysis, the macro PROCESS by Hayes was employed [32]. This macro is a path analysis modelling tool for the estimation of direct and indirect effects in mediation models. It is an empirical bias-corrected bootstrapping procedure for the estimation of confidence intervals from repeated resampling of the observed data. A mediation effect is significant only when the 95% confidence interval did not include zero. In this case, it would be concluded that in 95% of the bootstrapped samples the effect of resilience on HRQoL is mediated by the effect of autonomy (independence in ADLs and IADLs). In the present study, the data was resampled 10.000 times, as recommended by Hayes [32]. In small samples, bootstrapping has been demonstrated to be the most effective and powerful method to test indirect effects in comparison with other traditional methods, such as linear regression or the Sobel test. *p* < 0.05 was considered significant in all cases. All statistical analyses were conducted using SPSS, Version 24.0 (Armonk, NY, USA).

## 3. Results

### 3.1. Relationships between Resilience, ADLs, IADLs and HRQOL

Resilience showed a positive association with ADLs, IADLs and specific domains of HRQOL, such as Physical Function, Body Pain, Vitality, Social Function; and Physical Health Summary (for all <0.05). Regarding ADLs, this variable was significantly associated with HRQOL domains as Physical Function (*p* < 0.01), Role Physical (*p* < 0.05), Social Function (*p* < 0.05) and Physical Health Summary (*p* < 0.01). IADLs showed a significant relationship with Physical Function and Physical Health Summary (for all <0.01). The Pearson correlations between variables are summarized in Table 2.

### 3.2. Scores in ADLs and IADLs in Each Group

Differences between resilient and non-resilient groups in the domains of ADLs and IADLs were analysed (see Table 3). Regarding ADLs, significant differences between groups were found for Grooming (t(60) = −2.037, *p* = 0.046, d = 0.52), Bladder (t(60) = −2.200, *p* = 0.032, d = 0.56), Transfers (t(60) = −2.007, *p* = 0.049, d = 0.51) and Stairs (t(60) = −2.045, *p* = 0.045, d = 0.52). No significant differences were found in Feeding, Bathing, Dressing, Bowels, Toilet use and Mobility (*p* > 0.05). In the case of IADLs, differences between groups were found for Shopping (t(60) = −2.344, *p* = 0.022, d = 0.60), Food preparation (t(60) = −2.093, *p* = 0.041, d = 0.54), and Mode of transportation (t(60) = −3.283, *p* = 0.002, d = 0.84). In the case of Ability to Use Telephone, Housekeeping, Laundry, Responsibility for Own Medications and Ability to Handle Finances, no significant differences were found (*p* > 0.05). In all domains of ADLs and IADLs, resilient participants showed higher independence.

### 3.3. Scores in HRQOL in Each Group

**Figure 1:**
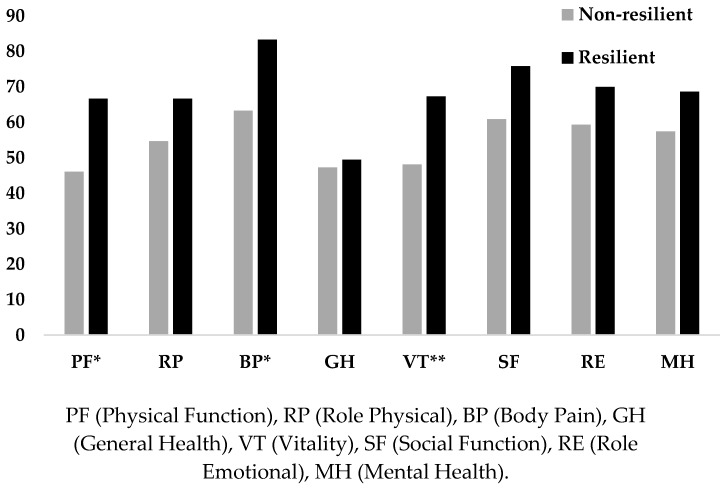
Differences in each domain of HRQOL between resilient and non-resilient participants (* *p* < 0.05; ** *p* < 0.01).

Differences between resilient and non-resilient participants in each domain of HRQOL were analysed. Significant differences were found in Physical Function (t(60) = −2.160, *p* = 0.035, d = 0.55), Body Pain (t(60) = −2.390, *p* = 0.020, d = 0.61) and Vitality (t(60) = −2.782, *p* = 0.007, d = 0.71). As can be observed in Figure 1, resilient participants exhibited a better HRQOL in comparison to low resilient ones. No significant differences were found in the other domains of HRQOL (*p* > 0.05).

### 3.4. Mediation Analyses

**Figure 2:**
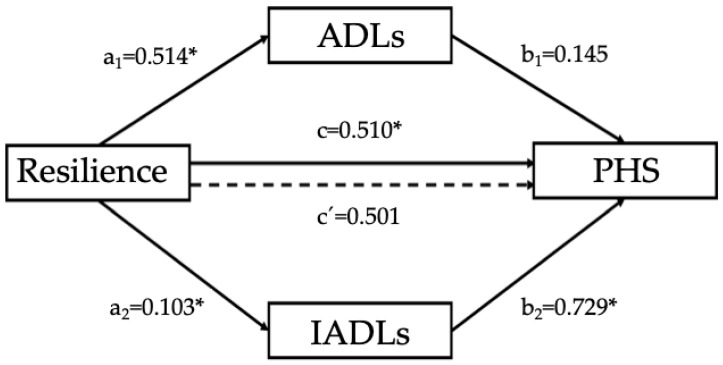
Representation of the relationships between the predicting variable, resilience, the mediator variables, ADLs and IADLs, and the criterion variable, PCS. The numerical values correspond to the unstandardized regression coefficients (* *p* < 0.05).

Multiple mediation analyses were conducted to evaluate the mediation effects of levels of dependence, measured by ADLs and IADLs, in the relationship between resilience and HRQOL, controlling for age, sex, marital status and cognitive functioning. Resilience was entered in the model as an independent variable, ADLs and IADLs as mediators and each summary component of the HRQOL (Physical Health Summary (PHS) and Mental Health Summary (MHS)) were evaluated as dependent variables in two models separately. No mediating effects were found with MHS as a dependent variable. However, when PHS was introduced in the model as a dependent variable, the mediation model was significant. First, resilience predicted ADLs (B = 1.06, SE = 0.514, *p* = 0.045) and IADLs (B = 0.211, SE = 0.103, *p* = 0.044). Regarding the mediator variables, only IADLs predicted PHS (B = 1.63, SE = 0.729, *p* = 0.028). The analysis of the indirect effect of resilience PHS, through the IADLs effect, showed a significant mediation (indirect effect = 0.347, bias-corrected 95% Confidence Interval for the indirect effect: lower level = 0.0015, upper level = 0.0765). When IADLs was introduced in the model as a mediator, the relationship between resilience and PHS did not show a statistically significant result (B = 0.625, SE = 0.501, *p* = 0.217), suggesting that IADLs have a full mediating effect in that association. Overall, the model (F(7,54) = 3.444, *p* = 0.004) predicted 31% of the variance in PHS in the participants. The covariates introduced in the final model revealed significance (Figure 2).

## 4. Discussion

The present study was aimed to understand the association between resilience coping, daily life functioning, evaluated through ADLs and IADLs, and HRQOL among older adults with MCI. Based on the obtained results, highly resilient coping is related to better performance in both ADLs and IADLs, and better HRQoL. Hence, when differences between resilient and non-resilient individuals were evaluated for specific basic and instrumental daily life activities and domains of HRQoL, significant differences were found. Specifically, those resilient individuals showed better performance in grooming, bladder control and mobility regarding ADLs, and in shopping, food preparation and use of means of transport in IADLs. In the case of HRQOL, resilient individuals exhibited higher scores in physical functioning and vitality and suffered from lower levels of body pain. In this sense, when ADLs and IADLs were tested as possible mediators in the relationship between resilience and physical component of HRQOL, IADLs were demonstrated to be a significant mediator in this association.

Although the relationship between resilience and health has been widely studied over the past few years [33,34,35,36,37,38], its influence in daily living functionality has been less analysed, especially in people with MCI. Previous studies have found that people with MCI suffer from a HRQoL reduction, with a significant impairment in levels of well-being [39,40,41,42,43]. It has been demonstrated that HRQoL is significantly impaired in people with MCI, in comparison to the general population [44]. This impairment involves several aspects of HRQoL that go beyond cognition, such as social relationship deterioration, reduction in the ability to enjoy himself/herself, and mood disturbances [44]. These negative consequences alongside the cognitive deficits of MCI could be directly related to the development of several levels of dependence in this population, entailing a reduction in their functionality and autonomy. However, based on the obtained results in the present study, higher levels of resilient coping could play a main role in this issue, improving the HRQoL by the maintenance of optimal levels of autonomy in people with MCI.

Resilience coping, as an ability to face several challenges of daily living, acquires higher importance in people with MCI, attending inherent cognitive impairment and its associated deficits [33,37]. One of the main mechanisms that could explain the relevance of resilience in this case is based on the compensatory mechanisms that resilient people could develop in order to reduce the impact of cognitive deficits [20]. From a classical perspective, the model of Selection, Optimization and Compensation (SOC) of Baltes and Baltes oriented to the explanation of successful aging takes into account the positive effects of compensation strategies to face losses associated with aging, maintaining a subjective sense of well-being [45]. From this perspective, successful aging is conceptualized as attending to three mechanisms that regulate the aging process: selection, optimization and compensation. Hence, the model conceptualizes processes that promote profits, thanks to selection and optimization, but also mechanisms that counteract losses inherent to the life cycle, through compensation of losses [45,46,47]. Selection becomes an adaptive mechanism as a result of the limitations of older adults and stimuli of the environment. Optimization is the process by which new resources and skills are acquired, ideas are copied or corrected from the resources of success that other people have, and their own energy is used to achieve personal goals. Finally, when resources are reduced or completely lost, compensation strategies, defined as a set of behaviours aimed at mitigating or adapting to loss [48], are necessary to avoid the reduction, modification or loss of resources relevant to the person [49]. These three mechanisms work as resources that allows coping with the problems that arise in life and act as protective factors in active aging [50]. For Baltes and Baltes [45], successful aging is a balance between gains and losses, so that in this process, people launch a series of psychological strategies by which they select what is important according to their needs and priorities, optimize the available resources and compensate for the losses inherent to the evolutionary moment to generate a development of life. In this way, people can compensate for losses and maintain a high level of satisfaction with their lives. Regarding people with cognitive deficits, it has been recently demonstrated that compensatory strategies that individuals perform in their daily living suppose an adaptive strategy to buffer the negative consequences of the cognitive decline [20]. Although older adults could develop compensatory strategies spontaneously, those resilient individuals could develop them consciously, and hence, maintain higher autonomy in daily living activities. In that respect, highly resilient individuals could be more prepared to select and optimize resources for the environment, and at the same time, implement compensatory strategies to face characteristic losses from aging. This fact is especially relevant in people with MCI, taking into account that they should face more significant and disabling losses than people with adequate cognitive functioning.

The positive relationships between resilience and activities of daily living found in the present study point out that resilient people with MCI develop greater compensatory resources, minimizing the impact of the loss of autonomy derived from cognitive deficits in day-to-day activities. Previous studies highlight this association in older people [51]. This fact is especially relevant regarding IADLs, taking into account that recent studies have demonstrated that individuals with MCI show a significant impairment in cognitively demanding instrumental activities of daily living (e.g., medication management and financial management) in comparison to healthy adults [52,53,54]. These activities, which require higher neuropsychological resources, seem to be most severely deteriorated than basic activities of daily living in individuals with MCI [52]. Hence, it has been demonstrated that the impairment of the functioning in IADLs may indicate an increased risk of progression to dementia in people with MCI [52]. Based on the results of mediation analyses, the greater autonomy in IADLs that show resilient individuals could have a positive effect in HRQoL, fundamentally in the case of a physical component. These results are in concordance with those obtained by Tomaszewsky et al. [20] in which greater frequency of compensation strategies was associated with higher levels of independence in everyday function, even after controlling for cognitive deterioration. 

Although the present research entails a significant advancement in the understanding of the protective effects of resilience buffering the negative impact of MCI in HRQOL, through the maintenance of higher levels of independence, mainly in IADLs, some limitations of the study should be addressed. First, due to the cross-sectional nature of the study, a causal relationship could not be established. Secondly, neither socioeconomic status or medication intake, variables that might influence HRQoL or autonomy in individuals with MCI, were not evaluated in the present study. Finally, the small size of the evaluated sample could limit the generalization of the obtained results. However, it should be taken into account that the whole sample is composed by people with MCI living in the community, which implies an ecological approach to the phenomenon. Future studies should investigate the specific influence of resilience on the level of autonomy of people with MCI employing longitudinal designs. Furthermore, attending that there is a specific classification of compensatory strategies to support memory loss [55], it is necessary that futures studies will evaluate the associations between resilience, autonomy and specific compensatory strategies in this population.

## 5. Conclusions

Overall, our results contribute to the existing body of literature, pointing out that resilient people with MCI exhibit higher autonomy in daily living activities, and, consequently, a greater HRQoL, fundamentally in the physical component. Resilience, as a coping style, could help people with MCI in finding and implementing the best compensatory strategies to face losses derived from cognitive deterioration. This fact entails a protective effect maintaining adequate levels of autonomy in activities of daily living, and hence, better HRQoL. These findings provide an important reference for policy health markers, researchers, therapists and community workers in their development of rehabilitation strategies for older people with MCI. Attending that resilience is an ability that can be worked on and developed in community samples of older adults [56], strengthening their capacity to adapt to the physical, psychological and social changes that affect over the years is extremely necessary. In this regard, it is necessary to include the evaluation of resilience levels in protocols of geriatric assessment in order to identify people with high risk of suffering a loss of autonomy and HRQoL deterioration due to their low levels of resilience. Based on this identification, and taking into account our findings, it would be crucial to work on resilience, especially in this group of low-resilient people with MCI in a preventive way, being able to act as a protective factor against the progression of functional deterioration. 

Future research could be oriented to the identification of other possible mediators, which could have a significant influence in the relationship between resilience and HRQoL in older people. Moreover, new researches should replicate the results obtained in the present study in other groups of people with cognitive impairment, such as individuals with dementia or people with subjective cognitive impairment. 

## Figures and Tables

**Table 1:** Mean and standard deviation, and frequency and percentage in sociodemographic characteristics and cognitive functioning in the whole sample—and each group.

Variable/Characteristics		All Sample *n* = 62	Non-Resilient *n* = 32	Resilient n = 30
**Sex**	**Male**	21 (33.9%)	14 (43.8%)	7 (23.3%)
	**Female**	41 (66.1%)	18 (56.2%)	23 (76.7%)
**Age**		77.52 ± 8.26	78.78 ± 8.22	76.17 ± 8.23
**Marital status**	**Single**	2 (3.2%)	1 (3.1%)	1 (3.3%)
	**Married**	27 (43.5%)	13 (40.6%)	14 (46.7%)
	**Divorced**	3 (4.8%)	1 (3.1%)	2 (6.7%)
	**Widowed**	30 (48.4%)	17 (53.1%)	13 (43.3%)
**Cognitive status**	**MMSE**	22.91 ± 2.21	23.09 ± 2.16	22.73 ± 2.30

**Table 2:** Patterns of correlations between resilience, ADLs, IADLs and components of HRQOL (* *p* < 0.05; ** *p* < 0.01).

Variable	Scale	1	2	3	4	5	6	7	8	9	10	11	12	13
	**1. Resilience**	1	0.279 *	0.320 *	0.314 *	0.096	0.318 *	0.137	0.316 *	0.249 *	0.075	0.201	0.287 *	0.144
	**2. ADLs**		1	0.595 **	0.466 **	0.275 *	0.185	0.097	0.138	0.310 *	0.039	0.149	0.399 **	0.030
	**3. IADLs**			1	0.548 **	0.153	0.204	0.001	0.185	0.127	−0.106	−0.038	0.460 **	−0.187
**HRQOL**	**4. Physical Function**				1	0.460 **	0.490 **	0.156	0.252 *	0.486 **	0.167	0.278 *	0.800 **	0.040
	**5. Role Physical**					1	0.419 **	0.360 **	0.312 *	0.584 **	0.471 **	0.532 **	0.609 **	0.427 **
	**6. Body Pain**						1	0.305 *	0.304 *	0.481 **	0.289 *	0.381 **	0.663 **	0.236
	**7. General Health**							1	0.407 **	0.358 **	0.082	0.273 *	0.507 **	0.188
	**8. Vitality**								1	0.378 **	0.102	0.447 **	0.363 **	0.435 **
	**9. Social Function**									1	0.246	0.525 **	0.572 **	0.483 **
	**10. Role Emotional**										1	0.597 **	−0.048	0.799 **
	**11. Mental Health**											1	0.153	0.869 **
	**12. Physical Health Summary**												1	−0.106
	**13. Mental Health Summary**													1

**Table 3:** Differences in each domain of ADLs and IADLs between resilient and non-resilient participants (* *p* < 0.05; ** *p* < 0.01).

Variable	Scale	Non-Resilient	Resilient	*p*
		*n* = 32	*n* = 30	
**ADLs**	**Feeding**	9.38 ± 1.68	9.50 ± 1.52	0.761
	**Bathing**	4.38 ± 1.68	4.83 ± 0.91	0.185
	**Dressing**	9.38 ± 1.68	9 ± 2.42	0.479
	Grooming *	4.38 ± 1.68	5 ± 0.00	0.044
	**Bowels**	10 ± 0.00	10 ± 0.00	-
	Bladder *	8.75 ± 2.84	10 ± 1.31	0.030
	**Toilet use**	9.69 ± 1.23	10 ± 1.31	0.337
	Transfers (bed to chair and back) *	12.97 ± 3.07	14.33 ± 2.17	0.047
	**Mobility (on levels surfaces)**	13.13 ± 3.30	14.20 ± 3.08	0.191
	Stairs *	6.56 ± 3.68	8.37 ± 3.22	0.045
**IADLs**	**Ability to use telephone**	0.94 ± 0.24	0.93 ± 0.25	0.948
	Shopping *	0.34 ± 0.48	0.63 ± 0.49	0.022
	Food preparation *	0.41 ± 0.49	0.67 ± 0.47	0.041
	**Housekeeping**	0.47 ±0.50	0.60 ± 0.49	0.308
	**Laundry**	0.50 ± 0.50	0.70 ± 0.46	0.111
	Mode of transportation **	0.34 ± 0.48	0.73 ± 0.45	0.002
	**Responsibility for own medications**	0.50 ± 0.50	0.73 ± 0.45	0.060
	**Ability to handle finances**	0.56 ± 0.50	0.70 ± 0.46	0.269
